# Matrix metalloproteinase inhibition mitigates renovascular remodeling in salt-sensitive hypertension

**DOI:** 10.1002/phy2.63

**Published:** 2013-08-22

**Authors:** Sathnur B Pushpakumar, Sourav Kundu, Naira Metreveli, Suresh C Tyagi, Utpal Sen

**Affiliations:** Department of Physiology and Biophysics, University of Louisville School of MedicineLouisville, Kentucky, 40292, USA

**Keywords:** Autophagy, collagen, Dahl salt sensitive, extracellular matrix, fibrosis, vascular density

## Abstract

Extracellular matrix (ECM) remodeling is the hallmark of hypertensive nephropathy. Uncontrolled proteolytic activity due to an imbalance between matrix metalloproteinases and tissue inhibitors of metalloproteinases (MMPs/TIMPs) has been implicated in renovascular fibrosis. We hypothesized that inhibition of MMPs will reduce excess ECM deposition and modulate autophagy to attenuate hypertension. Dahl salt-sensitive (Dahl/SS) and Lewis rats were fed on high salt diet and treated without or with 1.2 mg/kg b.w. of GM6001 (MMP inhibitor) by intraperitoneal injection on alternate days for 4 weeks. Blood pressure (BP), renal cortical blood flow, vascular density, collagen, elastin, and MMPs/TIMPs were measured. GM6001 treatment significantly reduced mean BP in hypertensive Dahl/SS rats. Renal resistive index (RI) was increased in hypertensive Dahl/SS rats and Doppler flowmetry showed reduced cortical perfusion. Barium angiography demonstrated a reduction in terminal branches of renal vasculature. Inhibition of MMPs by GM6001 resulted in a significant improvement in all the parameters including renal function. In hypertensive Dahl/SS rats, protein levels of MMP-9, -2, and -13 were increased including the activity of MMP-9 and -2; TIMP-1 and -2 levels were increased whereas TIMP-3 levels were similar to Lewis controls. Administration of GM6001 reduced the activity of MMPs and increased the levels of TIMP-1, -2, and -3. MMP inhibition reduced type 1 collagen deposition and increased elastin in the intrarenal vessels indicating reduced fibrosis. Autophagy markers were decreased in hypertensive Dahl/SS rats and GM6001 treatment enhanced their levels. We conclude that MMP inhibition (GM6001) reduces adverse renovascular remodeling in hypertension by modulating ECM turnover and stimulating autophagy.

## Introduction

Renovascular fibrosis is a hallmark of hypertensive nephropathy, which is associated with decreased tissue compliance and renal dysfunction. Current evidence implicates an imbalance of matrix metalloproteinases (MMPs) and their inhibitors, tissue inhibitors of metalloproteinases (TIMPs), as the key mediators in the fibrogenic process (Han et al. 2006; Catania et al. 2007; Castro et al. 2010). MMP participation results in remodeling of vascular, glomerular, and tubulointerstitial spaces by altering the turnover of extracellular matrix (ECM) proteins. The main structural alterations in the ECM include increased types I and III collagen and a greater collagen to elastin ratio which together confer increased resistance to volume flow (Funabiki et al. 1990; Intengan and Schiffrin 2001; Zhao et al. 2008).

Although MMPs are predominantly associated with matrix degradation, they have been shown to cleave bioactive molecules which may promote increased deposition of ECM proteins (Taipale et al. 1998; Yu and Stamenkovic 2000). The kidney expresses several MMPs/TIMPs some of which have been associated with diverse renal pathologies (Catania et al. 2007). Previous work in our laboratory demonstrated that increased MMP-2 and -9 in spontaneously hypertensive rats (SHR) contributed to significant glomerular injury and renal remodeling which was characterized by increased collagen deposition and elastinolysis (Camp et al. 2003). In a recent human study, elevated plasma MMP-9 level correlated with systolic BP, however, elevation of plasma MMP-2 and -10 had better correlation with hypertension-induced renal damage (Friese et al. 2009).

Congenic translocation of a segment of chromosome 10 from normotensive Lewis rats to hypertensive Dahl/SS rats has been shown to reduce high BP (Palijan et al. 2003). Using this model, our laboratory evaluated the relationship between MMPs and TIMPs on cardiac function in a previous study (Rodriguez et al. 2008). Hypertensive Dahl/SS rats exhibited left ventricular (LV) hypertrophy and dysfunction which was associated with increased expression of MMP-9 and reduced TIMP-4. In contrast, congenic animals showed normal BP and mitigation of LV remodeling associated with an increase in MMP-2 and TIMP-4 and a reduction in MMP-9 (Rodriguez et al. 2008). Whether the changes in the MMPs/TIMPs were the cause or effect of normalization in BP and thus LV changes were still not clear.

Normally, ECM homeostasis is maintained by a balance between synthesis and degradation of its proteins. This cellular regulatory mechanism is brought about by a process known as ‘autophagy’ in which cytosolic proteins are transferred to an autosome and then delivered to a vacuole containing lysozome for subsequent degradation (Yang and Klionsky 2009; Glick et al. 2010). A growing body of evidence now links autophagy to renal diseases such as acute kidney injury (AKI) (Jiang et al. 2010), polycystic kidney disease (Belibi et al. 2011), and diabetic nephropathy (Tanaka et al. 2012). These studies, however, are recent and provide only a general association of its involvement in various kidney diseases. Under normal conditions, the kidney is protected from the adverse effects of hypertension by endogenous regulators that include pressure natriuresis (Hall et al. 1986; Guyton 1991), antioxidants (Pedro-Botet et al. 2000), and prostaglandin E2 (Breyer and Breyer 2000). We believe that in addition to these mechanisms, autophagy may play a role in the maintenance of ECM. Earlier work in our laboratory has demonstrated that increased MMP-2, -9, and-13 activity was associated with interstitial fibrosis and right ventricular failure in a model of pulmonary artery constriction (Qipshidze et al. 2012). In the same study, autophagy markers were found to be elevated suggesting a concurrent mechanism causing mitochondrial dysfunction.

In this study, our aim was to determine whether inhibition of MMPs in hypertensive Dahl salt-sensitive rats would reduce excess protein deposition in the ECM and thus reduce renovascular fibrosis and hypertension. In addition, we also determined the potential role of autophagy as regulator of ECM homeostasis.

## Materials and Methods

### Animals and protocol

Animal protocols were performed in accordance with institutional animal care guidelines and conform to the *Guide for the Care and Use of Laboratory Animals* published by the U.S. National Institutes of Health (NIH Publication, 2011). Institutional Animal Care and Use Committee (IACUC) of the University Of Louisville School Of Medicine approved this study. Eight-week-old male Dahl salt-sensitive (Dahl/SS) and Lewis rats were purchased from Harlan Laboratories (Indianapolis, IN) and maintained on normal diet up to 6 months of age. They were then fed on a high salt diet (4% NaCl; Cincinnati Lab Supply, Cat. # 5882 C-5A) for 6 weeks. Tap water was provided ad libitum during the experiment. After 6 weeks of high salt diet, the animals were divided into four groups: Two groups of Dahl/SS (*n* = 5/group) and Lewis (*n* = 6/group) received vehicle alone (0.9% NaCl in water [w/v]) and two other groups of Dahl/SS and Lewis rats received a MMP inhibitor (GM6001) for 4 weeks. GM6001 dissolved in DMSO was further diluted with vehicle and given at a dose of 1.2 mg/kg b.w. on alternate days by intraperitoneal injections. BP was measured by noninvasive tail-cuff method (CODA; Kent Scientific, Torrington, CT). Animals were placed on a warming platform and allowed to acclimatize for 10 min before measurements were taken. Baseline BP was recorded before starting animals on high salt diet and repeated every fortnight thereafter. At the end of treatment, laser Doppler flowmetry was performed under intraperitoneal pentobarbital anesthesia. After blood collection, animals were euthanized with an overdose of pentobarbital injection followed by barium angiography and tissue harvest. Renal function was assessed by measuring plasma creatinine according to the manufacturer's instructions with Quantichrome Creatinine assay kit (DICT-500; BioAssay Systems, Hayward, CA).

### Antibodies and reagents

Rabbit polyclonal antibodies for MMP-2, -9, -13, TIMP-1, -2 and -3, and anti-GAPDH were purchased from Millipore (Temecula, CA). Ilomastat (GM6001; Cat # CC 1010) was purchased from Millipore (Billerica, MA). Horseradish peroxidase-linked anti-rabbit IgG antibody was from Santa Cruz Biotechnology (Santa Cruz, CA).

### Renal ultrasonography for blood flow and resistive index

Renal ultrasonography was performed before commencement of salt treatment and at the end-point of the experiment. Animals were anesthetized by isoflurane inhalation and placed supine on a heated table. Body temperature was maintained at 37.5°C. After depilation, acoustic gel (Other-Sonic; Pharmaceutial Innovations, Newark, NJ) was applied on the skin and imaging was performed using Vevo 2100 system (VisualSonics, Toronto, ON, Canada). The transducer, MS250 (13–24 MHz), was held immobile by an integrated rail system (VisualSonics) during imaging. The kidney was scanned in the long and short axis. All measurements were done on the left side and included renal artery diameter, peak systolic, and end-diastolic blood flow velocity (mm/sec) in the renal artery and cortex by Pulsed-Wave Doppler mode. Cine loops were exported and analyzed to obtain resistive and pulsatility index.

### Laser Doppler cortical blood flow measurement

Under intraperitoneal pentobarbital anesthesia, the animal was placed in right lateral position and the left kidney was exposed through a paraspinal longitudinal incision. Renal cortical blood flow was measured using Speckle Contrast Imager (Moor FLPI, Wilmington, DE) at room temperature. The camera (580 × 752 resolution) was positioned 15 cm from the dorsal surface of kidney; settings for low-resolution/high-speed images included a display rate of 25 Hz, time constant of 1.0 sec, and camera exposure time of 20 msec. The contrast images were processed to produce a color-coded live flux image and a flux units trace was also recorded for 2 min in all the animals.

### Barium angiography for renal vascular density

Barium sulfate (0.1 g/mL) was suspended in 50 mmol/L Tris buffer (pH 5.0) and infused 1 mL volume at a constant pressure and flow with a syringe pump through a cannula PE10 (ID – 0.28 mm, BD, Franklin Lakes, NJ) introduced via the infrarenal aorta/renal artery. The left kidney was first flushed with 0.9% saline followed by perfusion of barium contrast. Animals were placed in the X-ray chamber and angiograms were captured with Kodak in vivo imaging system FX Pro (Molecular Imaging System; Carestream Health Inc., Rochester, NY). Two-minute X-ray images were taken at 35 Kvp. Vessel was quantified in two steps by the software developed by University of Lubeck (http://www.isip.uni-luebeck.de/?id=150). First, the barium image was enhanced by converting it to gray scale and inverted to show vessels against a distinct background. This was followed by final automatic segmentation avoiding any false-positive errors. The percentage of white pixels (vessels) was calculated against the number of pixels in the background by the program automatically to give the vascular percentage.

### Histological analysis

Hematoxylin and eosin stain was performed on 5-μm-thick kidney sections for microscopy and measurement of wall-to-lumen ratio in intrarenal vessels. Collagen deposition was measured by using Picrosirius red stain kit following manufacturer's instructions (Polysciences, Inc., Warrington, PA) with modification. After deparaffinization and hydration, 5-μm-thick kidney sections were incubated in picrosirius red solution overnight, followed by 0.01 N HCl treatment for 2 min and dehydrated again. Slides were mounted and three to five high-power field images were captured under polarized light filter microscope (Olympus FluoView1000; B&B Microscope Ltd, Pittsburg, PA). ImagePro Plus software (Media Cybernetics, Inc., Rockville, MD) was used to quantify types I and III collagen in the renal cortical vessels as the proportion of yellow and green colored areas, respectively.

Elastin staining was performed on 5-μm-thick sections after deparaffinization and hydration. Sections were stained with Chromaview™ Elastin Stain Kit (Richard-Allan Scientific, Kalamazoo, MI) following manufacturer's instructions. Briefly, sections were immersed in freshly prepared Verhoeff's Iron Haematoxylin solution for 15–20 min, rinsed in water, differentiated in working ferric chloride solution until sections appeared dark brown. They were then washed under running tap water for 2–3 min and stained with Van Gieson stain solution for 1 min. After dehydration and clearing, slides were mounted using permount mounting media (Fisher Scientific, Pittsburgh, PA). For image analysis, three to five high-power images were captured under light microscope (Olympus FluoView1000; B&B Microscope Ltd) and quantified using ImageJ software (National Institute of Health [NIH]).

### Western blot analysis

Whole kidney tissues were homogenized with RIPA buffer (Boston BioProducts, Inc., Ashland, MA) containing Phenylmethanesulfonyl fluoride and protease inhibitor cocktail (Sigma-Aldrich, St. Louis, MO) and the protein concentration was measured by Bradford method. Samples at equal concentration and volume were electrophoresed in sodiumdodecyl sulphate-polyacrylamide gel electrophoresis (SDS-PAGE) at 70 V, transferred onto PVDF membranes at 120 mA overnight. Membranes were blocked for 1 h with 5% nonfat milk in Tris Buffered Saline with Tween 20 at room temperature and incubated with appropriate primary antibody at 4°C overnight. Respective Horseradish Peroxidase-conjugated secondary antibody incubation was for 2 h at room temperature. After washing, membranes were developed using chemiluminescence (Pierce ECL western blotting substrate; Thermo Scientific, Rockford, IL). GAPDH was used as loading control and band intensity was quantified using ImageJ software.

### SDS-PAGE zymography

Equal concentration and volume of kidney samples were minced in ice-cold extraction buffer (1:3 w/v) containing 10 mmol/L cacodylic acid, 20 mmol/L ZnCl_2_, 1.5 mmol/L NaN_3_, and 0.01% Triton X-100 (pH 5.0) and incubated overnight at 4°C with gentle agitation. The homogenate was centrifuged for 15 min at 1500 g and the supernatant collected. Protein concentration in the sample was measured using Bradford method, and 50 μg of the protein was electrophoretically resolved for each sample in 10% SDS-PAGE containing 0.1% gelatin as MMP substrate. Gels were washed in renaturing buffer with one change in between to remove SDS, rinsed in water, and incubated for at least 48 h in developing buffer at 37°C in a water bath with gentle shaking. Gels were stained with 0.5% Coomassie brilliant blue. MMP activity in the gel was detected as white bands against a dark blue background. Band intensity was quantified using ImageJ software.

### Statistical analysis

All values are presented as the mean ± standard error of the mean (SEM). Statistical analysis was performed using Primer of Biostatistics (Ver. 7; McGraw-Hill, Blacklick, OH). Multiple comparisons were analyzed using analysis of variance followed by Bonferroni's post hoc test. Mann–Whitney rank sum test was performed for ordinal data. The threshold for significance was *P* < 0.05.

## Results

### Effect of MMP inhibitor (GM6001) on hypertension and renal function

**Figure 1:**
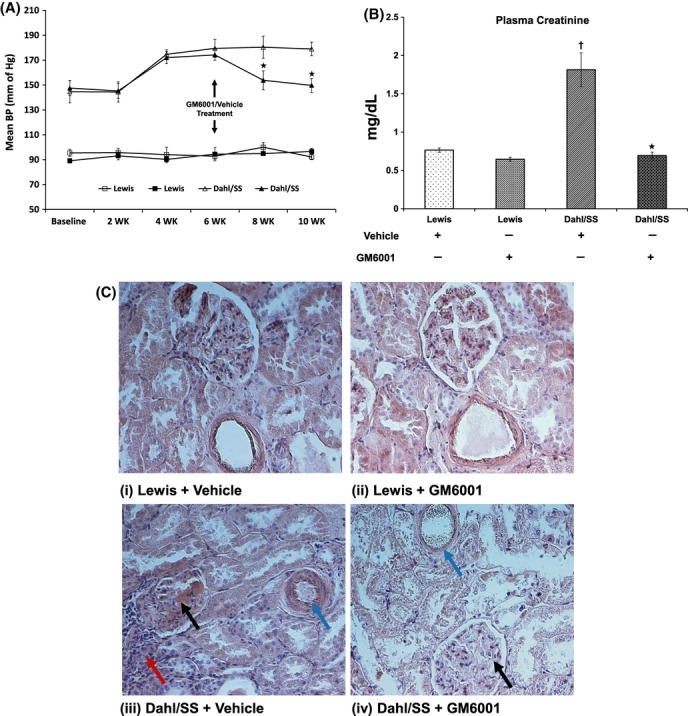
(A) Mean arterial blood pressure (BP) measurement from Lewis (*n* = 6/group) and Dahl/SS rats (*n* = 5/group) on 4% salt diet. BP was measured using the tail-cuff method. GM6001, a nonspecific inhibitor of MMP was administered after 6 weeks of salt diet and continued until end of experiment. Hollow symbols (△,□) represent vehicle treatment and solid symbols (▲,▪) represent GM6001 treatment. *P < 0.04 versus Dahl/SS + vehicle. (B) End-point plasma creatinine levels in Lewis and Dahl/SS rats after 6 weeks of high salt diet followed by administration of a nonspecific MMP inhibitor, GM6001. Each experiment was performed in duplicate and values presented as mean ± SEM. (C) Representative images using H&E staining: (i) and (ii) Sections of kidney tissue from of Lewis groups (Vehicle and GM6001) demonstrating normal glomeruli, tubules, and vessels, (iii) Dahl/SS rats treated with vehicle only showing glomerular protein (black arrow), narrowing of vessel lumen (blue arrow) and interstitial inflammation (red arrow), (iv) Dahl/SS rats treated with GM6001 showing normal glomerulus (black arrow) and vessel diameter (blue arrow). *P < 0.05 versus Dahl/SS + vehicle, ^†^*P* < 0.05 versus Lewis groups (Vehicle and GM6001). MMPs, matrix metalloproteinases.

Dahl salt-sensitive (Dahl/SS) rats had a higher baseline mean arterial pressure (MAP) than Lewis controls on normal diet (Fig. 1A). On high salt diet, MAP in Dahl/SS rats increased after 2 weeks reaching a plateau at 4 weeks which was maintained till the end of the experiment. In contrast, Lewis rats maintained their MAP similar to their baseline values (Fig. 1A). In Dahl/SS animals, MAP decreased 2 weeks after treatment with intraperitoneal GM6001 whereas no effect was observed in Lewis groups without or with MMP inhibition. Hypertensive Dahl/SS rats had poor renal function as indicated by high plasma creatinine levels which was increased by more than twofold compared to Lewis rats. Treatment with MMP inhibitor (GM6001) diminished and normalized creatinine levels in Dahl/SS rats, but had no effect on Lewis rats (Fig. 1B).

### Renal resistive index, cortical blood flow, and vascularity

**Figure 2:**
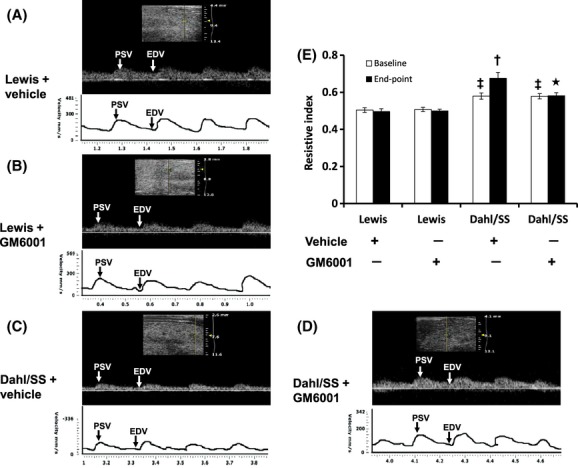
Renal ultrasound analysis in Lewis and Dahl/SS rats. Kidney ultrasound was measured using the Visualsonics Vevo Ultra imaging system as described in Materials and Methods. Top panel A–D: Doppler spectral image in gray scale from one experiment, bottom panel of A–D: Line drawing of Doppler spectral images. PSV: peak systolic velocity (mm/sec); EDV: end-diastolic velocity (mm/sec). Values are represented as resistive Index (RI) (E). RI measures the resistance of renal arterial flow to the kidney which is calculated as (peak systolic velocity – end-diastolic velocity)/peak systolic velocity. Values are from four individual experiment and presented as mean ± SEM. *P < 0.05 versus. Dahl/SS + vehicle control, ^†^*P* < 0.05 versus baseline in Dahl/SS (Vehicle); ^‡^*P* < 0.05 versus Lewis groups (Vehicle and GM6001).

Renal ultrasound was performed to detect changes in blood flow in the renal artery and cortical vessels of the kidney. RI is a measurement of the degree of vascular resistance and thus an indirect measurement of blood flow. Chronic hypertension affects resistance vessels in the kidney to increase RI. In Dahl/SS rats, RI increased significantly compared to Lewis rats (Fig. 2). Upon treatment with GM6001, RI returned to baseline values comparable with Lewis rats. There was no change in RI of Lewis rats without or with GM6001 treatment (Fig. 2).

**Figure 3:**
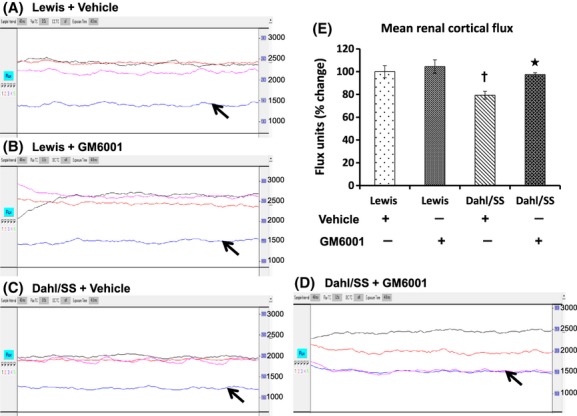
Renal cortical blood flow measurement of left kidney in Lewis and Dahl/SS rats on high salt diet and treated with vehicle and GM6001. A–D: The traces represent flux units (No. of RBCs × velocity) in aorta (black trace), renal artery (red trace), renal cortex (blue trace), and renal vein (purple trace) from one experiment. Bar graph (E) represents the percent change in the mean flux units in the renal cortex ± SEM from five individual experiments in each group. *P < 0.05 versus Dahl/SS + Vehicle, ^†^*P* < 0.05 versus Lewis.

To further quantify the blood flow in the renal cortex, we used Laser Doppler flowmetry. Our results indicated a significant reduction in the renal cortical blood flux units (No. of RBC × velocity) in vehicle-treated Dahl/SS rats compared to Lewis rats (Fig. 3). Renal perfusion was normalized with GM6001 treatment. No differences in blood flux units were observed in Lewis rats treated without or with GM6001 (Fig. 3).

**Figure 4:**
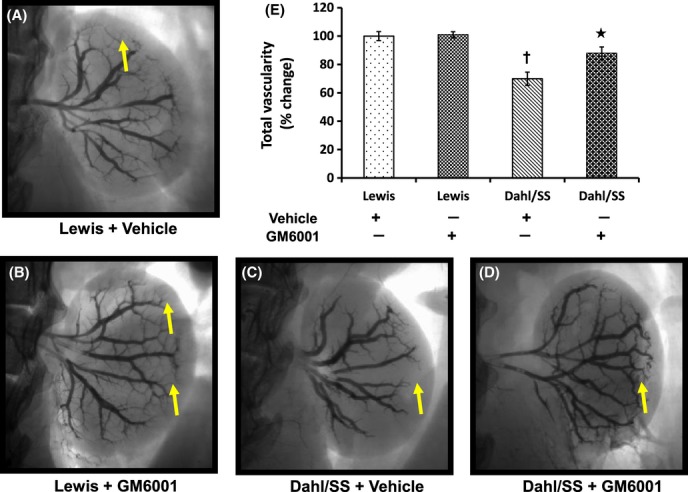
Barium angiogram of left kidney in Lewis and Dahl/SS groups (vehicle and GM6001) (A–D). To visualize renal vascular architecture, 1.0 mL of barium sulfate (0.1 mg/mL) was infused in a retrograde fashion via infrarenal aorta at constant pressure and time. Yellow arrows show the presence or absence of terminal branches in the renal cortex. Bar diagram (E) represents the mean percent change in renal vessel coverage against the background ± SEM (*n* = 5/group). *P < 0.05 versus Dahl/SS + Vehicle, ^†^*P* < 0.05 versus Lewis (Vehicle and GM6001).

Renal vascular density was measured by Barium angiography. In Lewis groups (vehicle and GM6001), renal angiography demonstrated normal vasculature including the terminal arcuate and interlobular (Fig. 4A,B) branches. Hypertensive Dahl/SS rats showed a significant reduction in arcuate and interlobular branches indicating vascular collapse (Fig. 4 C, yellow arrow) and thus decreased cortical blood perfusion (Fig. 3C). GM6001 treatment improved vascular density in Dahl/SS (Fig. 4D), particularly of the arcuate arteries (Fig. 4D, yellow arrow).

### Renal histology for collagen–elastin remodeling in renal vasculature and wall-to-lumen ratio

H&E staining revealed normal glomeruli and intrarenal vessels in Lewis groups treated with vehicle and GM6001. In contrast, hypertensive Dahl/SS rats showed protein deposition in the glomeruli, vascular constriction, and interstitial inflammation (Fig 1C(iii), black, blue, and red arrows, respectively). In Dahl/SS rats treated with GM6001, glomerular pathology was mitigated along with restoration of normal lumen diameter (Fig 1C(iv), black and blue arrows, respectively).

**Figure 5:**
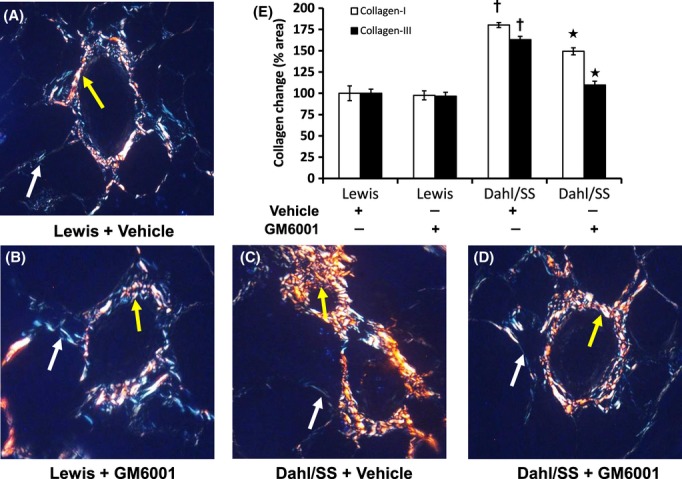
Images of intrarenal vessels captured with polarized filter. Type I (yellow) and type III (bluish green) collagen deposition in the renal resistance vessels were quantified using ImageJ software. Yellow and white arrows show areas of collagen types I and III, respectively. A–D: 5-μm paraffin sections were stained with picrosirius red staining kit as described in Materials and Methods. E: Bar graph represents collagen I (hollow) and collagen III (solid) as mean percent change in area in the intrarenal vessels ± SEM (*n* = 5/group). *P < 0.05 versus Dahl/SS + Vehicle, ^†^*P* < 0.05 versus Lewis (Vehicle and GM6001). Original magnification ×60.

Picrosirius red-stained kidney sections were visualized under polarized light filter to determine the levels of types I and III collagen. In Figure 5, type 1 collagen is represented by yellow color and type III as green color. Type I collagen was predominant in the vessels and type III in the tubules in both Dahl/SS and Lewis rats. There was no difference in the levels of types I and III collagen in the vessels or tubules of Lewis rats without or with GM6001 (Fig. 5). Type I collagen was higher in the vessels of Dahl/SS kidney compared to Lewis rats and GM6001 treatment significantly reduced its content. The areas of type III collagen in the vessels were also increased in vehicle-treated Dahl/SS rats, which were mitigated by GM6001 treatment.

**Figure 6:**
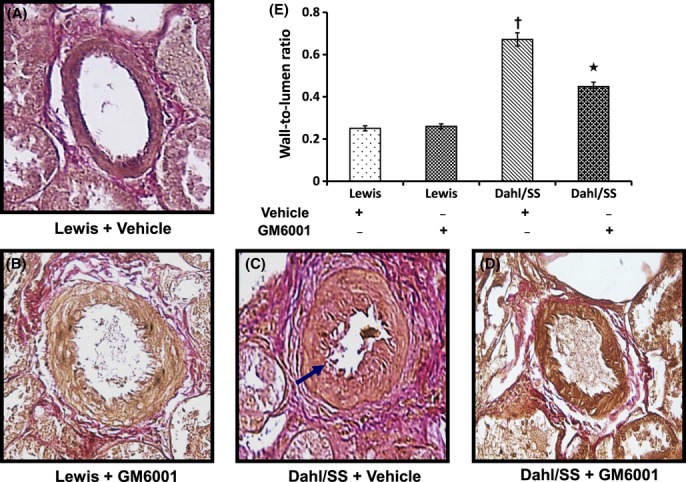
Elastin content in the tissue was measured in 5-μm-thick sections with Van Gieson's stain (A–D). Images A–D represent interlobular arteries in the renal cortex. Hypertensive Dahl/SS rats show increased wall thickness, elastin disruption, and narrowing of lumen diameter (blue arrow). Bar diagram (E) shows mean wall thickness–lumen diameter ratio ± SEM from five individual experiments. *P < 0.05 versus Dahl/SS + Vehicle; ^†^*P* < 0.05 versus Lewis (Vehicle and GM6001). Image magnification ×60.

Renal vessels in Lewis rats showed normal patency and the total elastin content remained unchanged without or with GM6001 treatment (Fig. 6A,B). In contrast, Dahl/SS rats on vehicle treatment showed a decrease in elastin with extensive disruption and severe narrowing of the lumen in the terminal vascular branches (Fig. 6C). GM6001 treatment maintained the integrity of elastin layers and together by decreasing collagen normalized the diameter of vessel lumen (Fig. 6D).

The wall-to-lumen ratio was measured to indicate the severity of arteriosclerosis in the intrarenal vessels. There was no difference in this ratio in Lewis groups treated without or with GM6001 (Fig. 6A,B,E). In contrast, Dahl/SS rats treated with vehicle showed a significantly higher ratio than in animals receiving GM6001 treatment (Fig. 6C–E).

### Hypertension increases renal expression and activity of MMP-9 and -2

**Figure 7:**
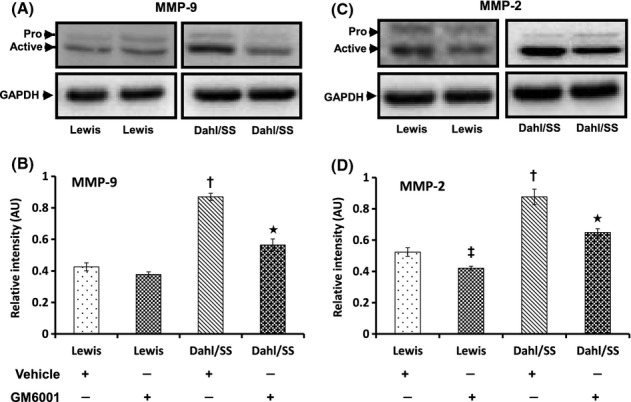
Renal expression of MMP-9 and MMP-2 was increased in hypertensive animals. Fifty micrograms of protein from each group was separated by SDS-PAGE and immunoblotted using rabbit anti-MMP-9 (A) and anti-MMP-2 (C) antibodies. The pixel densities of bands from five experiments were quantified using ImageJ software [National Institute of Health (NIH)] and presented as mean relative intensity ± SEM (B and D for MMP-9 and -2, respectively). *P < 0.05 versus Dahl/SS + Vehicle; ^†^*P* < 0.05 versus Lewis (Vehicle and GM6001); ^‡^*P* < 0.05 versus Lewis treated with Vehicle. MMPs, matrix metalloproteinases, SDS-PAGE, sodiumdodecyl sulphate-polyacrylamide gel electrophoresis.

We measured the expression and activity of gelatinases as they are the key factors involved in ECM remodeling. The expression of both metalloproteinases was diminished in Lewis groups (vehicle and GM6001) (Fig. 7A–D). Dahl/SS rats treated with vehicle showed increased expression of MMP-9 (Fig. 7A,B) and -2 (Fig. 7C,D) compared to Lewis controls (Fig. 7A–D, respectively). A significant reduction in MMP-2 was also observed in Lewis rats following treatment with GM6001 (Fig. 7B,D).

**Figure 8:**
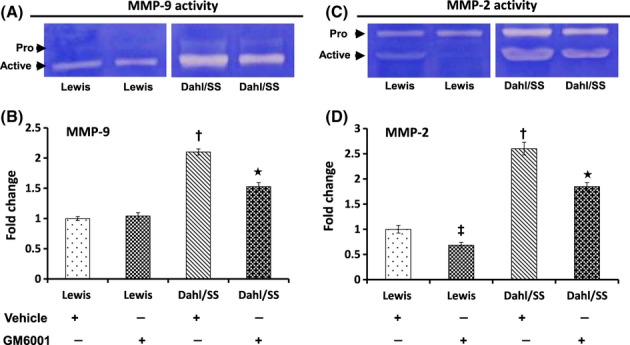
Gelatin zymography showing increased gelatinolytic activity of MMP-9 and MMP-2 (A and C, respectively). Zymography was performed using 0.1% gelatin gel as described in Materials and Methods followed by Coomassie blue staining. Bar diagrams represent fold change of MMP-9 and MMP-2 (B and D, respectively) considering the control value as 1. Values are presented as mean ± SEM from five individual experiments. *P < 0.05 versus Dahl/SS + Vehicle, ^†^*P* < 0.05 versus Lewis (Vehicle and GM6001); ^‡^*P* < 0.05 versus Lewis treated with vehicle. MMPs, matrix metalloproteinases.

To determine whether the expression of MMP-9, -2 corroborated with their activities, we performed in gel gelatin zymography. The activity of MMP-9 and -2 was significantly increased in Dahl/SS rats on vehicle treatment which was mitigated in GM6001-treated Dahl/SS animals (Fig. 8A,C). In Lewis rats, the activity of MMP-9 did not differ between vehicle and GM6001 treatment (Fig. 8A,B); however, the activity of MMP-2 was reduced with GM6001 (Fig. 8C,D).

### Expression of MMP-13 and TIMP-1 and -2 is increased in hypertension

**Figure 9:**
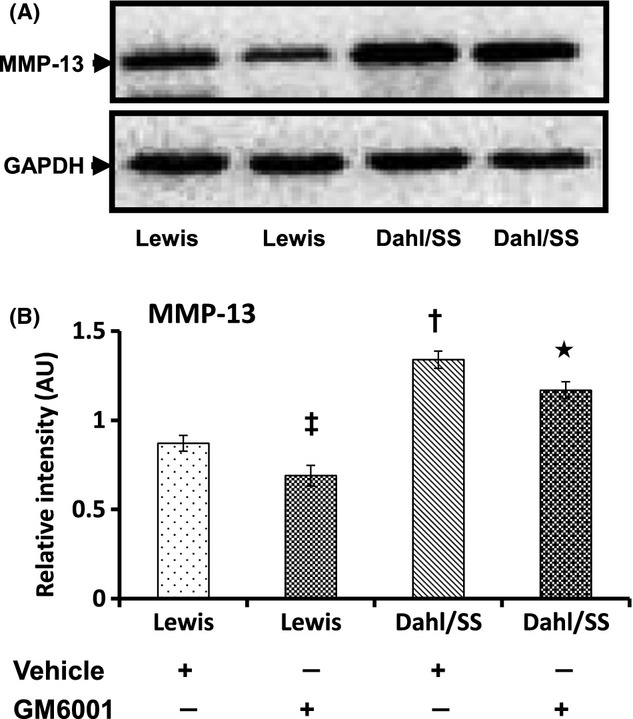
Expression of MMP-13 was increased in Dahl/SS + Vehicle-treated rats (A). Fifty micrograms of protein from each group was separated by SDS-PAGE and immunoblotted using rabbit anti-MMP-13. Bar diagram (B) shows the mean intensity ± SEM. *P < 0.05 versus Dahl/SS + Vehicle; ^†^*P* < 0.05 versus Lewis groups; ^‡^*P* < 0.05 versus Lewis treated with Vehicle. MMPs, matrix metalloproteinases; SDS-PAGE, sodiumdodecyl sulphate-polyacrylamide gel electrophoresis.

**Figure 10:**
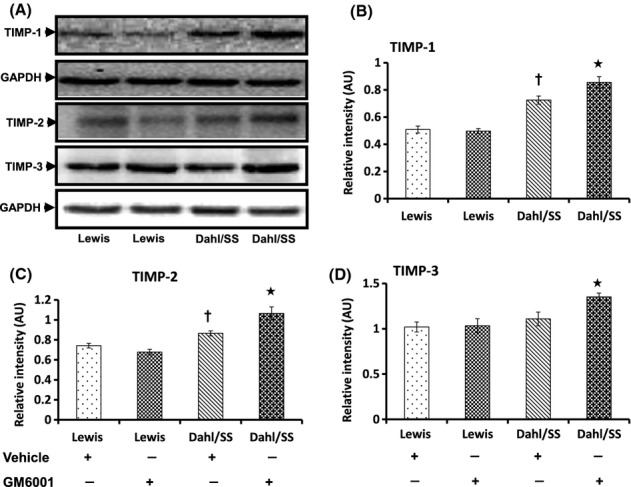
Hypertensive Dahl/SS rats showing increased expression of TIMP-1 and -2 and normal levels of TIMP-3 compared to Lewis control animals (A). The bar diagrams (B, C, and D) show mean relative intensity ± SEM of four separate experiments. *P < 0.05 versus Dahl/SS + Vehicle, ^†^*P* < 0.05 versus Lewis (Vehicle and GM6001). TIMPs, tissue inhibitors of metalloproteinases.

The deposition of interstitial collagen types I and III contributes to fibrosclerosis in the kidney. We therefore measured the expression of collagenase MMP-13 and the inhibitors TIMP-1, -2, and -3. As expected, MMP-13 was increased in hypertensive Dahl/SS rats, whereas treatment with GM6001 decreased its expression (Fig. 9). TIMP-1 expression was similar in vehicle- and GM6001-treated Lewis groups (Fig. 10A,B). In hypertensive Dahl/SS rats, TIMP-1 levels were increased compared to Lewis rats (vehicle and GM6001) which increased further following GM6001 treatment (Fig. 10A,B). TIMP-2 expression showed a similar increase in hypertensive Dahl/SS rats treated with vehicle and reached even higher levels after GM6001 treatment (Fig. 10A,C). The expression of TIMP-3 was similar in Lewis groups (Vehicle and GM6001) and vehicle-treated Dahl/SS rats but increased in Dahl/SS rats receiving GM6001 (Fig. 10A,D).

### Matrix metalloproteinases inhibition stimulates autophagy in hypertensive rats

**Figure 11:**
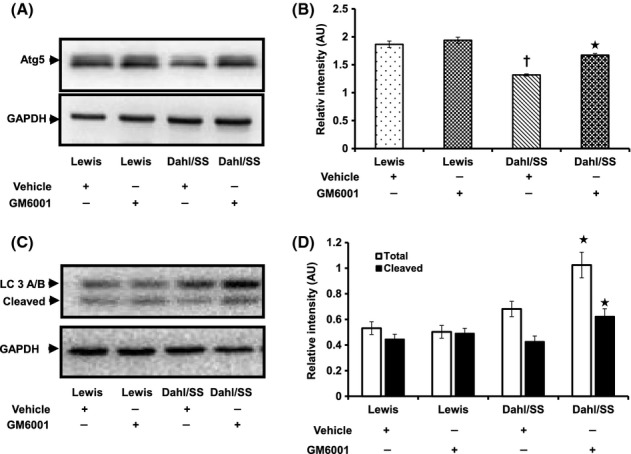
Autophagy is impaired in hypertensive Dahl/SS rats. Fifty micrograms of protein was loaded in each well and probed with Atg5 (A) and LC3A/B (C) antibodies. Values are expressed as mean intensity ± SEM from five individual experiments. (B): *P < 0.05 versus Dahl/SS + Vehicle, ^†^*P* < 0.05 versus Lewis (Vehicle and GM6001); (D): *P < 0.05 versus Dahl/SS + Vehicle.

Atg and LC3 are proteins which upon conjugation form complexes that are involved in the formation of autophagophore and hence serve as autophagosomal markers. We measured Atg5 and LC3A/B to determine whether hypertension modulated autophagy in the kidney. In Lewis rats, Atg5 levels were similar without any significant difference between vehicle and GM6001 treatment (Fig. 11A,B). In contrast, Dahl/SS rats treated with vehicle showed reduced Atg5 indicating decreased autophagy (Fig. 11A,B). LC3A/B is a variant of LC3, a microtubule-associated light-chain protein which is an important component of autophagy. During autophagy, LC3A/B-1 undergoes posttranslational modification to yield LC3A/B-II in a process involving Atg7 and Atg3 to form autophagic vacuoles. Similar to Atg5, the expression of LC3A/B-II was decreased in Dahl/SS rats receiving vehicle-only treatment (Fig. 11C,D). GM6001 treatment increased the expression of Atg5 and LC3A/B in Dahl/SS rats suggesting stimulation of autophagy.

## Discussion

In the current study, we demonstrate that hypertensive Dahl/SS rats sustain significant renal injury and undergo extensive renovascular remodeling. In addition to MMP-2 and MMP-9, we demonstrate that MMP-13 also plays a significant role in hypertension-induced renal injury. ECM changes are predominant in the renal resistance vessels which showed increased type 1 collagen than type III indicating vascular stiffness and thus reduced compliance. Inhibition of MMPs using GM6001 modulated ECM changes to normalize BP and also reduced renovascular fibrosis. We also found that MMP inhibition was associated with increased levels of autophagy markers suggesting a parallel mechanism regulating ECM homeostasis.

GM6001 is a broad-spectrum MMP inhibitor that mediates its action by binding to active zinc sites present on the MMPs (Jones et al. 1997). Previously, GM6001 has been shown to induce a variety of effects including improvement of vascular function in preeclampsia (Abdalvand et al. 2013), prevention of agonist-induced vessel remodeling (Martinez-Lemus et al. 2011), and in reduction of MMP-induced vasoconstriction in cardiac microvessels of SHR (Rodrigues et al. 2010). In our study, although GM6001 did not attenuate high BP in Dahl/SS rats completely, there was a significant reduction compared to untreated controls suggesting that MMP activity, in part, may be contributing to altered vascular tone in hypertension. In addition, GM6001 has also been shown to improve renal function by reducing blood urea nitrogen in cisplatin-induced injury (Ramesh and Reeves 2004). We, however, chose to measure plasma creatinine as this is more reflective of glomerular function and show that GM6001 normalized creatinine levels indicating better renal function. A recent study evaluated the effect of two new compounds, XL081 and XL784 (MMP inhibitors) in hypertensive Dahl/SS rats and Diabetic T2DN rats in reducing renal pathology (Williams et al. 2011). Although XL081 and XL784 are described as specific inhibitors of MMPs, their inhibitory activity extends to other MMPs (Williams et al. 2011). In the study above, both XL081 and XL784 reduced the development of proteinuria and glomerulosclerosis, but only XL081 decreased the mean BP whereas the more potent MMP inhibitor, XL784, had no effect. In our study, the reduction in mean BP by GM6001 was similar to XL081 and further, remodeling in intrarenal vessels was significantly decreased. The reduction in BP by MMP inhibitors with overlapping activities across other MMPs as in the above studies including our own suggests a more complex role of several MMPs in hypertensive nephropathy and renovascular remodeling. Although GM6001 reduced the activity of MMPs in this study, the exact mechanism by which it decreases the expression of MMPs is not clear and warrants further investigation.

Doppler RI is a noninvasive method used to quantify alterations in renal hemodynamics and is a measure of resistance offered by renal vessels. Although an increase in RI may occur due to a combination of lesions in the glomeruli, tubulointerstitium, and vessels, studies have shown that they correlate better with renal arteriosclerosis (Ikee et al. 2005; Bige et al. 2012). In fact, intimal thickening and a reduction in lumen diameter were the key factors contributing to an increase in the RI (Bige et al. 2012). Our data from ultrasonography and Doppler flowmetry suggest a decrease in compliance and together with increased plasma creatinine (Fig. 1B) indicates significant renal damage. The reduction in the terminal arcuate and interlobular branches in hypertensive rats seen on angiography is possibly due to these vessels being subjected to extensive remodeling causing luminal narrowing and eventual collapse. Inhibition of MMP with GM6001, by reducing the intrarenal resistance (Fig. 2D,E), improved regional blood flow (Fig. 3D,E) and preserved renal function (Fig. 1B).

The activity of MMPs control the structural alteration in collagen and elastin during hypertensive remodeling (Ishii and Asuwa 2000; Galis and Khatri 2002). MMP-2 and -9 are produced by the majority of cells types found in the kidney and have a substrate specificity for collagen types III and IV and elastin (Ahmed 2009). Collagen types I and III are predominant in the vasculature and interstitium (Bishop and Lindahl 1999; Zeisberg et al. 2002) and collagen IV in the glomerular basement membrane. In a recent report, increased deposition of collagen and elastin has been reported as early as 2 weeks in aorta in renovascular hypertension (Ceron et al. 2012). In the same study, the authors show that early in hypertension this is mainly due to increased MMP activity and over time the levels of MMPs increase further contributing to progressive accumulation of ECM proteins. Chamiot et al. reported that subtypes of collagen are also an important determinant of large artery stiffness (carotid and aorta). In their study, they found that Japanese strain of SHR, when compared to Lyon strain, showed lesser aortic hypertrophy and increased elastin which was associated with increased density of collagen type III but not collagen type I (Chamiot Clerc et al. 1999). In contrast, we observed increased type I collagen than type III in the intrarenal arteries of hypertensive rats (Fig. 5) which suggested that collagen remodeling may vary in different organs and strains. MMP-13 is a collagenase expressed in the glomerulus with substrate specificity for types I, II, and III collagen (Tomita et al. 2004; Ahmed 2009). There is scant literature regarding its role in causing renal injury. In a murine study, the absence of MMP-13 protected the mice from Adriamycin-induced renal injury suggesting a pathogenic role in this model (Sakamaki et al. 2010). Another study defining the role of cytokine cardiotrophin 1, in cardiac, vascular, and renal function found increased renal fibrosis with cardiotrophin 1 infusion which was associated with a decreased MMP-13/TIMP-1 ratio suggesting a protective role in this model (Lopez-Andres et al. 2012). Our findings suggest that in addition to MMP-2 and -9, MMP-13 is also likely to regulate ECM remodeling in hypertension. A significant reduction in their levels following the administration of GM6001 further strengthens their participation in renovascular fibrosis. GM6001 has also been reported to possess elastase inhibitory activity (Cowan et al. 2000), which may explain the maintenance of elastin integrity in intrarenal vessels seen in our study (Fig. 6D,E).

Although the renal expression of different TIMPs is species dependent, rat kidneys express TIMP-1, -2, and -3 in abundance (Catania et al. 2007). A high expression of TIMP-1 in SHR was shown to increase Col1a1 deposition and development of fibrosis (Hultstrom et al. 2008). Similarly, in unilateral urethral obstruction model, persistent elevation of TIMP-1 expression resulted in fibrosis (Duymelinck et al. 2000). A study conducted by Kim et al., however, found that genetic deficiency of TIMP-1 alone along with higher MMP-9 levels did not decrease the severity of renal fibrosis. They speculate that this may be due to compensation by other protease inhibitors (Kim et al. 2001). In this study, we observed increased levels of TIMP-1 and -2 in hypertensive rats with no change in TIMP-3 (Fig. 10). A further increase in their levels following treatment with GM6001 suggests the involvement of complex regulatory mechanisms in renovascular fibrosis which warrants further studies.

Autophagy is a highly conserved cellular process which is now recognized to play a dual role in cell survival as well as cell death under conditions of stress (Baehrecke 2005; Periyasamy-Thandavan et al. 2009). A pathogenic role for autophagy was first demonstrated by Chien et al. (2007)who showed increased beclin-1 and LC3 markers in kidneys following ischemia–reperfusion injury. Also, in vitro experiments in renal proximal tubular cells showed that increased autophagy turnover contributed to increased cell death during hypoxic conditions (Suzuki et al. 2008). In contrast to the above studies, deficient autophagy has been implicated in the pathogenesis of diabetic nephropathy (Tanaka et al. 2012), cisplatin-induced renal injury (Yang et al. 2008; Jiang et al. 2012), tubular injury following sepsis (Hsiao et al. 2012), and acute ischemic injury (Kimura et al. 2011). Defective autophagy has been shown to promote intracellular accumulation of oxidized proteins from damaged cell organelles leading to glomerular injury in aging mice (Hartleben et al. 2010). Indeed, such protein aggregates has also been implicated in neurodegenerative diseases (Mizushima et al. 2008). Renovascular fibrosis in hypertension may, in part, be due to defective autophagy and impaired clearance as evidenced by collagen accumulation seen in our study. This is in agreement with a report by Kim et al. (2012) who showed that deficiency of autophagy protein beclin-1 in mice promoted increased deposition of collagen in the kidneys. Similarly, inhibition of autophagy has been associated with ECM production of fibroblasts and development of idiopathic pulmonary fibrosis (Patel et al. 2012). We also found that treatment with GM6001 increased the levels of Atg5 and LC3A/B suggesting increased autophagy which was accompanied by increased collagen degradation, preservation of elastin, and normalization of luminal diameter.

Limitations: Our results show an improvement in BP and other renovascular parameters following GM6001 treatment in established hypertension; whether similar treatment would prevent the development of hypertension or its structural changes is not known. Although a change in the levels of TIMPs was observed following GM6001 treatment, the specific role of TIMPs in salt-sensitive hypertension cannot be concluded with the present findings without the use of specific inhibitors. As glomerular and tubulointerstitial pathologies can also worsen hypertension, further studies are required to identify the specific effects of GM6001 on these individual factors. Finally, although we show deficient autophagy contributing to hypertensive changes in the kidney, the site, that is, glomerulus, tubules or the vessels and the exact mechanisms involved are still to be elucidated.

In conclusion, we show that renovascular fibrosis due to hypertension can be attributed in part, to increased expression and activity of MMP-9, -2, and -13. In addition, this is the first study to demonstrate a role for autophagy in hypertension-induced renal remodeling. Inhibition of MMP and upregulation of autophagy may therefore have potential for therapeutic application to attenuate high BP and renal remodeling in hypertensive nephropathy.
